# Gut Microbiota as a Trigger for Metabolic Inflammation in Obesity and Type 2 Diabetes

**DOI:** 10.3389/fimmu.2020.571731

**Published:** 2020-10-16

**Authors:** Torsten P. M. Scheithauer, Elena Rampanelli, Max Nieuwdorp, Bruce A. Vallance, C. Bruce Verchere, Daniël H. van Raalte, Hilde Herrema

**Affiliations:** ^1^Department of Internal Medicine, Amsterdam University Medical Center (UMC), Vrije Universiteit (VU) University Medical Center, Amsterdam, Netherlands; ^2^Department of Experimental Vascular Medicine, Amsterdam University Medical Center (UMC), Academic Medical Center, Amsterdam, Netherlands; ^3^Division of Gastroenterology, Department of Pediatrics, Child and Family Research Institute, Vancouver, BC, Canada; ^4^Department of Surgery, University of British Columbia and BC Children's Hospital Research Institute, Vancouver, BC, Canada

**Keywords:** microbiota, obesity, metainflammation, metabolism, diabetes

## Abstract

The gut microbiota has been linked to the development of obesity and type 2 diabetes (T2D). The underlying mechanisms as to how intestinal microbiota may contribute to T2D are only partly understood. It becomes progressively clear that T2D is characterized by a chronic state of low-grade inflammation, which has been linked to the development of insulin resistance. Here, we review the current evidence that intestinal microbiota, and the metabolites they produce, could drive the development of insulin resistance in obesity and T2D, possibly by initiating an inflammatory response. First, we will summarize major findings about immunological and gut microbial changes in these metabolic diseases. Next, we will give a detailed view on how gut microbial changes have been implicated in low-grade inflammation. Lastly, we will critically discuss clinical studies that focus on the interaction between gut microbiota and the immune system in metabolic disease. Overall, there is strong evidence that the tripartite interaction between gut microbiota, host immune system and metabolism is a critical partaker in the pathophysiology of obesity and T2D.

## Introduction

Type 2 diabetes (T2D) incidence, which is in large driven by the obesity pandemic, is increasing with alarming rates. In 2019, it was estimated that 463 million people were suffering from diabetes worldwide; these numbers are expected to continue to rise toward 578 million patients in 2030 (1). T2D is typically preceded by insulin resistance; a condition in which the actions of insulin on peripheral tissues including skeletal muscle, liver, and adipose, are impaired. This results in reduced insulin-stimulated glucose disposal, impaired insulin-induced suppression of hepatic glucose production and lipolysis rates, respectively (2). Insulin is produced by the beta cells of the endocrine pancreas. In early stages of insulin resistance, the pancreas can compensate for impaired peripheral insulin action by increasing insulin production. When pancreatic beta cells fail to meet the increased insulin demand, hyperglycemia develops (3). Hyperglycemia has been extensively linked to the detrimental micro- and macrovascular complications typically observed in humans with T2D (4).

**Figure 1:**
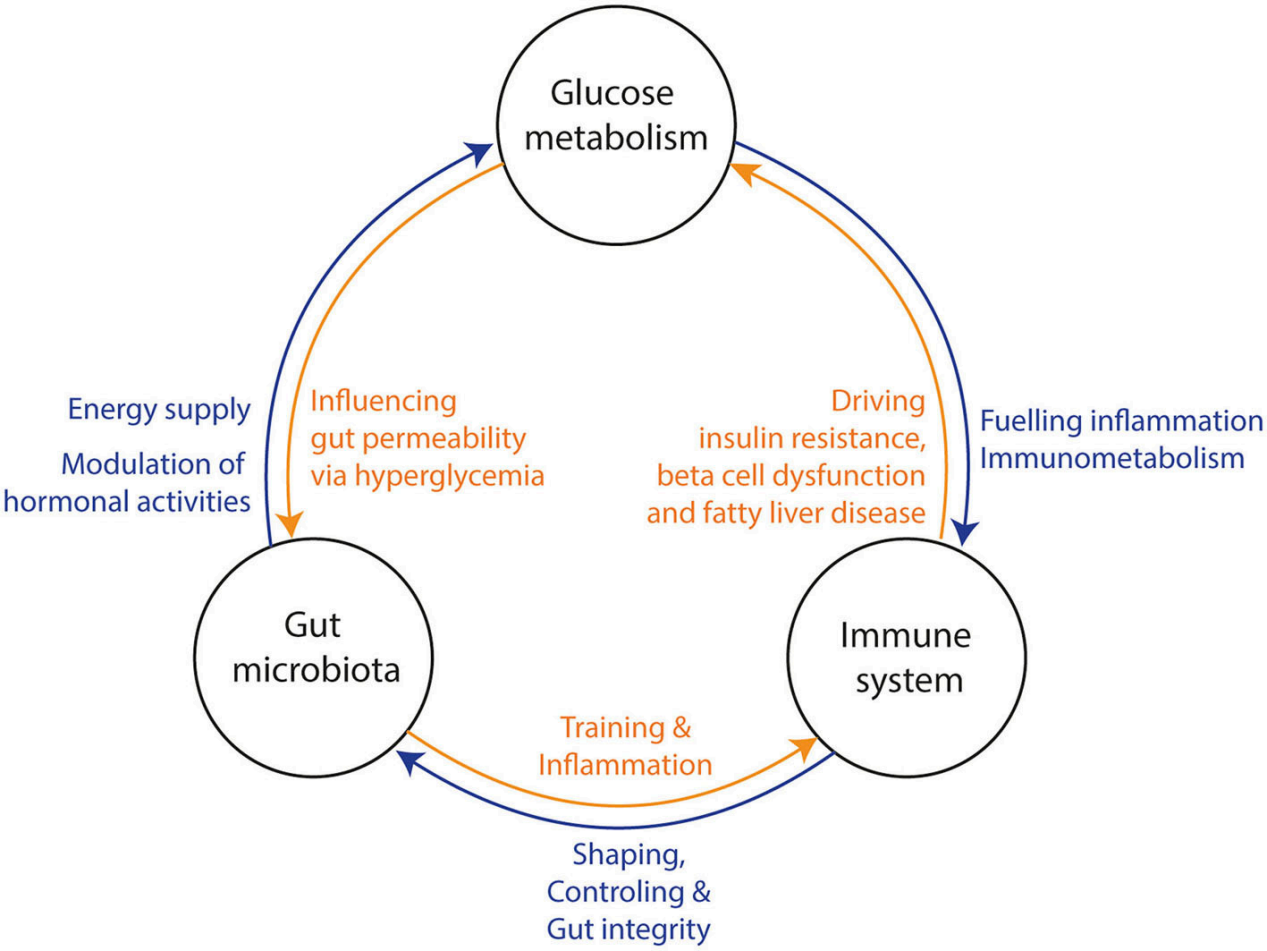
Three-way interaction between the gut microbiota, glucose metabolism, and the immune system. (1) The gut microbiota influences the host‘s glucose metabolism and hormone production via the production of several metabolites. Hyperglycemia increases gut permeability and thereby translocation of bacterial components into the circulation. In turn, bacterial translocation is fueling a (pro) inflammatory response of the immune system. Under normal conditions, the gut microbiota is training the immune system via several bacterial components and metabolites. (2) The immune system is shaping and controlling gut microbiota to keep a symbiotic relationship between host and microbiota. Further, it prevents bacterial translocation via promoting gut integrity. Bacterial translocation may lead to inflammation in several tissues and consequential loss of function (e.g., beta-cell dysfunction, insulin resistance and fatty liver disease). (3) The glucose metabolism can induce a pro-inflammatory response of the immune system through interplay of metabolic and inflammatory pathways (immunometabolism). Thereby, all three factors affect each other and may drive metabolic diseases.

In recent decades, chronic low-grade “metabolic” inflammation (local and systemic), also called metainflammation, has been identified to contribute to the development of insulin resistance and progression to T2D. As such, metainflammation has been linked to both impaired insulin action and secretion (5). While a vast body of research has provided detailed insight into regulation of glucose homeostasis by inflammatory pathways, the upstream triggers of these pathways have remained elusive for a long time. The intestinal microbiota, the collective community of microorganisms in the gastrointestinal tract, plays a critical role in human metabolism, in part by acting as an immunomodulator. Although this property is critical for human health, it can also have detrimental consequences. In accordance, the gut microbiota has been appointed as driver of metainflammation observed in obesity and T2D, which are also characterized by an altered gut microbiota composition (6–8). In this review, we extensively address the tripartite interaction between the gut microbiota, the mammalian immune system and glucometabolic pathways (Figure 1). In addition, we propose whether, based on current evidence, modulation of inflammation via the intestinal microbiota could form a target for novel therapies to reduce the current diabetes pandemic.

## Inflammation in Obesity and Type 2 Diabetes

**Figure 2:**
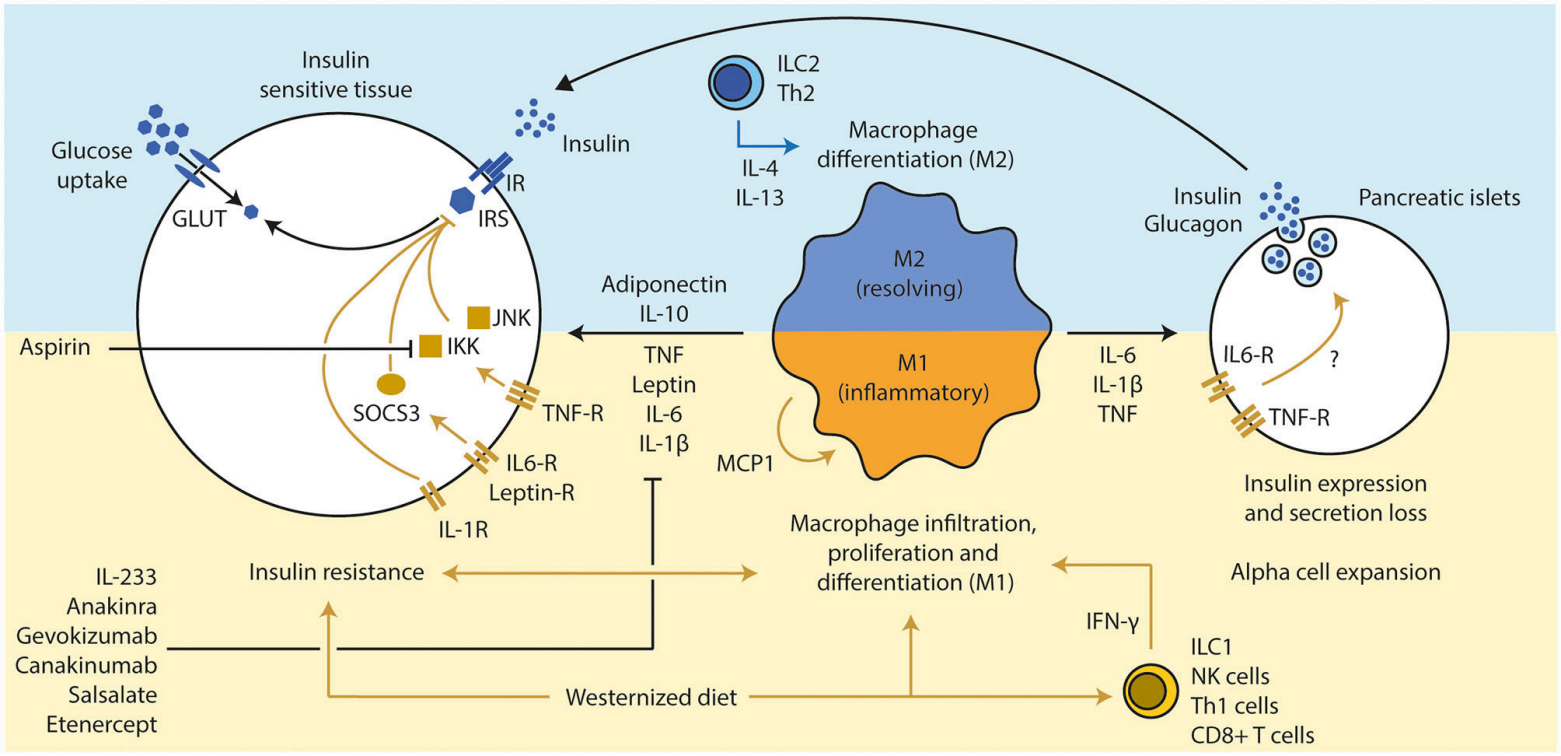
Inflammation influences beta cell function and insulin sensitivity. (1) A westernized diet induces insulin resistance and a pro-inflammatory immune response in metabolic active tissues. T cells (Th1 via IFN-γ and CD8+ T cells) have been discussed a secondary mediator that led to the attraction of macrophages, which are the main source of several pro-inflammatory cytokines. (2) An active pro-inflammatory response in those tissues enhances and deteriorates the extend of the insulin resistance via several inflammatory mediators (TNF, IL-6, and IL-1β), mainly secreted from M1 macrophages. Several downstream molecules (JNK, IKK, and SOCS3) interfere with the insulin signaling. Inhibition of those pro-inflammatory pathways led to improvement of insulin sensitivity and glucose tolerance (e.g., pharmacological treatments such as anakinra, gevokizumab, and aspirin). Anti-inflammatory cytokines such as IL-10 (expressed by various immune cell types, but mainly M2 macrophages) and adiponectin (from adipocytes) can resolve inflammation and improve insulin sensitivity. (3) Chronic high concentrations of pro-inflammatory cytokines lead to alpha cell expansion and beta cell dysfunction in pancreatic islets, which drives the progression toward T2D in obese subjects. Th, T helper cell; IFN, interferon; CD, cluster of differentiation; TNF, tumor necrosis factor; IL, interleukin; JNK, c-Jun N-terminal kinases; IKK, IκB kinase; SOCS, suppressor of cytokine signaling; GLUT, glucose transporter; IR, insulin receptor; IRS, insulin response substrate; MCP, monocyte chemoattractant protein 1.

Low-grade inflammation has been extensively linked to disturbances in glucometabolic pathways as observed in people with obesity and T2D. Several cytokines are increased in the circulation of people with metabolic syndrome (9–11) and have negative effects on peripheral tissue metabolism. For example, people with insulin resistance and glucose intolerance had a higher inflammatory tone and an altered response to respiratory viral infections compared to insulin sensitive individuals (12). Similarly, obese subjects had a higher inflammatory tone and more severe asthma, which was related to the gut microbiota (13). Interestingly, a 10% weight loss reduced plasma concentrations of several cytokines in obese women (10). Further, numerous pharmacological treatments aiming to reduce inflammation in metabolic diseases have positive effects on glucose tolerance in mice and human. These include the novel hybrid cytokine interleukin (IL) 233, which is produced in a genetically modified *Escherichia coli* by fusing murine IL-2 and murine IL-33 (14). Other treatment strategies include IL-1 receptor blockage (15), IL-1β antagonism (16), inhibition of the intracellular pro-inflammatory NF-κB pathway (17), and TNF antagonism (18) (Figure 2). These findings highlight pivotal roles of metainflammation in the development of T2D and the opportunities for (pharmacological) interventions. Below, we review a number of key immune cell types and inflammatory mediators that have been linked to impaired glucose metabolism.

Adipose tissue was one of the first tissues in which inflammation was highly correlated with insulin resistance. It contains a plethora of immune cells including T cells, eosinophils and mast cells that keep resident macrophages in an M2 polarized or alternatively activated state (19). Although these terms have fallen out of favor due to the identification of various other macrophage subtypes (20), we will stick to these terms for the sake of simplicity. Importantly, secretion of the anti-inflammatory cytokine IL-10 from M2 macrophages has been shown to protect lean mice from insulin resistance (21, 22). Obese mice and people with T2D have lower IL-10 expression and higher pro-inflammatory signals (21, 23). Obese humans with metabolic syndrome had lower levels of IL-10 compared to subjects without metabolic syndrome (24). Further, IL-10 overexpression in murine muscle tissue improved insulin sensitivity even under high fat diet conditions (22). These findings suggest a pivotal role of IL-10 in prevention of inflammation in people with metabolic abnormalities.

Other important, but less studied, anti-inflammatory cytokines include IL-4 and IL-13. IL-4 promotes glucose tolerance and inhibits adipogenesis (25). Further, it promotes an alternative macrophage activation (26, 27). Genetic variations in the IL-4 promotor have been associated with T2D susceptibility (28). However, the literature is scarce to draw conclusions. IL-13 shares a similar structure to IL-4, has anti-inflammatory properties (29) and promotes an alternative macrophage activation (30). Surprisingly, both cytokines were elevated in the blood circulation of obese humans compared to lean controls, which was associated with a lower physical activity (31). Another study supported this findings by showing increased levels in insulin resistant humans, which positively correlated with hyperglycemia (32). A study in mice points toward a disturbed IL-13 receptor activity (32). Obese animals have higher IL-13Rα2 activity, which inactivates and depletes IL-13. Therefore, the anti-inflammatory characteristics of IL-13 might be abolished despite high circulating levels. More research is necessary about anti-inflammatory cytokines in metabolic diseases.

M1 or classically activated macrophages have particularly been implicated in the metainflammation observed in metabolic diseases (33). M1 macrophages are generally responsible for secretion of pro-inflammatory cytokines and are associated with T2D development by altering local and distant tissue functions.

Certain cytokines (called chemokines) are able to attract immune cells to metabolic active tissues (34). Monocyte chemoattractant protein-1 (MCP1) is a strong chemokine for monocytes (35), and its expression was increased in obese human (36) and rodent adipose tissue (37, 38). In rodents, MCP1 overexpression in adipose tissue increased macrophage infiltration and mediated insulin resistance, whereas MCP1 knock out in combination with high fat diet feeding augmented development of insulin resistance compared to wild type mice (38). Although this observation was not observed in another study (39), higher chemokine production in obese adipose tissue has been associated with infiltration of immune cells and development of insulin resistance.

A recent study suggests that obesity-related insulin resistance precedes the infiltration of pro-inflammatory macrophages, giving insights in the cause and consequence dilemma (40). First, insulin resistance, which was genetically induced by knocking out mammalian target of rapamycin complex 2 (mTORC2), was shown to coincide with increased MCP1 expression, monocyte infiltration and differentiation into pro-inflammatory macrophages (M1). Second, insulin resistance in wild type mice preceded the accumulation of macrophages during diet-induced obesity. Thirdly, adipose tissue from obese insulin resistant patients had lower mTORC2 signaling, high MCP1 expression and high macrophage content (40). Thereby, insulin resistance might be the consequence of obesity and the cause for macrophage infiltration, which in turn amplifies the development toward diabetes.

Human pancreatic islets express and secrete MCP1 as well. MCP1 expression was increased after exposure to pro-inflammatory stimuli such as lipopolysaccharide (LPS) (41). Furthermore, overexpression of MCP1 in murine pancreatic beta cells led to robust macrophage infiltration in islets and spontaneous development of diabetes (42). These results suggest a major role of MCP1 in the pathogenesis of insulin resistance and beta cell dysfunction via immune cell infiltration.

Cytotoxic CD8 T cell infiltration into epididymal adipose tissue of obese mice precedes accumulation of macrophages. In fact, genetic depletion of CD8+ T cells lowered macrophage infiltration, adipose tissue inflammation and insulin resistance, whereas adoptive transfer of CD8+ T cells aggravated adipose inflammation (43). Further, T cells in obese adipose tissue produce more pro-inflammatory mediators compared to lean controls (44, 45). Moreover, the number of interferon (IFN) γ producing T helper (Th) 1 cells in human visceral adipose tissue positively correlates with systemic inflammatory tone, but are not associated with insulin resistance; though, the anti-inflammatory Th2 cells negatively correlated with insulin resistance (46). Further, obese mice lacking IFN-γ display lower adipose tissue inflammation and better glucose control (47, 48). Therefore, an interplay between different immune cells takes place, which has to be further eluted.

Innate lymphoid cells (ILCs) are recognized as the innate counterpart of T cells due to similar functionality (49). However, they miss the adaptive antigen receptors of T cells (50). They are divided into 5 subsets: Natural killer (NK) cells, ILC1, ILC2, ILC3, and lymphocytes tissue-inducer cells (LTi) (51). ILCs protect barrier tissues against pathogens and maintain immune homeostasis in several tissue types (52). Further, some of the ILCs have cytotoxic characteristics that are important to remove transformed cells and keep macrophages in homeostasis (51). Under steady state, cytotoxic ILCs kill adipose tissue macrophages to maintain homeostasis (53). However, this is impaired under high fat diet conditions leading to an increase of pro-inflammatory macrophages in the adipose tissue of obese mice and humans.

High fat diet increases NK cell numbers and the production of pro-inflammatory TNF in epididymal adipose tissue (54). Depletion of NK cells decreased adipose tissue macrophages, inflammation, and insulin resistance (54). Further, high fat diet drove the proliferation of ILC1 in adipose tissue and promoted a pro-inflammatory environment for macrophages via IFN-γ secretion (52). Similarly, obese subjects had higher ILC1 counts in adipose tissue and blood, which decreased after bariatric surgery (55). Therefore, NK cells and ILC1 seem to contribute to obesity phenotype by promoting a pro-inflammatory environment.

Obesity increased the activity and proliferation of NK cells, which stimulated the production of IFN-γ (56). That in turn stimulated the differentiation of macrophages and promoted insulin resistance (56). In contrast, high fat diet reduced the numbers of ILC2s in adipose tissue (57). They are important to sustain metabolic homeostasis in adipose tissue (58) and to keep macrophages in a M2 phenotype (57). IFN-γ suppressed the activity of ILC2s (58). Collectively, several recent studies indicate the involvement of ILCs in (adipose tissue) inflammation; potentially posing the unknown immunological trigger that induces a pro-inflammatory environment. However, the ILC research is relatively young to draw major conclusions yet.

Tumor necrosis factor (TNF), which is mostly expressed and secreted by adipose tissue macrophages (59), was one of the first cytokines shown to be increased in adipose tissue (60) and circulation of people with T2D and obesity (61). TNF expression in adipose tissue was inversely correlated to insulin sensitivity in obese people without T2D compared to heathy lean controls (62). Interestingly, TNF infusion in rats disturbed insulin sensitivity already on day one (63). Further, mice deficient in TNF and TNF receptor 1 & 2 were protected from diet-induced insulin resistance (64). Neutralization of TNF by infusing reactive immunoglobulins in rats improved insulin-stimulated glucose uptake (60). Lastly, weight loss in obese subjects improved insulin sensitivity and reduced TNF expression in adipose tissue (65), suggesting a pivotal role of obesity on inflammation and insulin sensitivity. TNF inhibition has been suggested for the treatment and prevention of T2D (66, 67). However, large and long-term clinical studies are warranted.

Several studies addressed the mechanism behind the insulin disturbing effects of TNF (68). TNF activates the intracellular IκB kinase (IKK), which comprises two kinases (alpha and beta), leading to the activation of NF-κB (nuclear factor kappa-light-chain-enhancer of activated B cells) (69) and transcription of inflammatory genes. Interestingly, inhibition of IKKβ by the anti-inflammatory agent aspirin or sodium salicylate (70) increased insulin sensitivity in mice (71) and humans (72, 73). Further, TNF is able to activate c-jun N-terminal kinase (JNK) (74), which is a direct inhibitor of the insulin signaling pathway (75). JNK activity was elevated in several tissue types of obese mice (76). Absence of JNK1 resulted in decreased adiposity and improved insulin sensitivity in mice (76–78). Both downstream molecules (IKK and JNK) of TNF interfere with insulin receptor substrate 1 (IRS1) and thereby stop insulin signaling (69, 79). Furthermore, within pancreatic islets, TNF production by macrophages drives dysfunction of insulin-producing beta cells and may directly mediate insulin resistance of pancreatic beta cells (80, 81). Therefore, TNF plays a crucial role in the development of insulin resistance in several tissue types with high glucometabolic relevance.

Adipose tissue macrophages are the main source of the highly studied pro-inflammatory cytokine IL-6, with estimated contributions of 15–35% of the total circulating IL-6 (59, 82). Mohamed-Ali et al. (82) measured the IL-6 concentration in arterial as well as venous blood from subcutaneous adipose tissue. They found two times more IL-6 in the venous blood compared to arterial blood, indicating major portions of circulating IL-6 is secreted from the adipose tissue. Surprisingly, they found no increase in TNF between both vessel types. Further, Weisberg et al. (59) confirmed that most of the IL-6 (and TNF) secreted from adipose tissue is originating from macrophages. Chronically elevated levels of IL-6 have been shown to decrease hepatic insulin sensitivity *in vitro* (83), to induce hyperinsulinemia in mice (84) and to mediate insulin resistance in murine muscle tissue (85). Mechanistically, IL-6 increases the activity of suppressor of cytokine signaling 3 (SOCS3), which inhibits several downstream mediators of insulin receptor signaling (86–88). Interestingly, knocking out the receptor for IL-6 in immune cells did not protect from insulin resistance, but disturbed immune homeostasis in mice (89, 90), suggesting a fine-tuned mechanism.

In the pancreas, the IL-6 receptor is mostly expressed in the endocrine portion, with higher levels in alpha cells (91). IL-6 expression was increased in pancreatic islets from obese mice (92) and associated with expansion of alpha cells, a known histological observation in the islets of people with T2D. Indeed, IL-6 knock out in obese mice inhibited the expansion of alpha cells, accompanied by reduced glucose stimulated insulin secretion (GSIS) (91). In addition, IL-6 administration in mice enhanced insulin secretion by increasing the incretin glucagon-like peptide 1 (GLP1) from intestinal L cells and pancreatic alpha cells (93, 94). Therefore, IL-6 has physiological effects on pancreatic islets, but the underlying mechanism is yet to be revealed.

The pro-inflammatory cytokine IL-1β has been implicated in the development of obesity and T2D (95–97). IL-1 receptor knockout mice are protected against high-fat diet induced glucose intolerance and adipose tissue inflammation (98). However, IL-1β has been shown to have physiological roles in glucose metabolism. Feeding increased IL-1β secretion from peritoneal macrophages in a glucose depended manner, which contributed to postprandial insulin secretion. Lack of endogenous IL-1β reduced postprandial insulin (99). Therefore, although IL-1β plays a physiological role in glucose metabolism, chronically elevated levels might lead into T2D.

In T2D, pancreatic islets are infiltrated by pro-inflammatory macrophages (92, 100), which drive the production of IL-1β (101) via the NLRP3 (NACHT, LRR, and PYD domains-containing protein 3) inflammasome (102). Initially, IL-1β at low concentrations may be beneficial by promoting β-cell proliferation (103); however, chronically elevated concentrations might lead to beta cell failure (66, 104). Administration of an IL-1 receptor antagonist improved glucose tolerance via improving beta-cell function and systemic inflammation in humans (15).

Further, chronic IL-1β administration is able to induce insulin resistance in adipose tissue *in vitro* (105). The mechanisms by which IL-1β mediates insulin-resistance have been attributed, at least in part, to downregulation of insulin substrate receptor-1 (IRS-1) (95) and aberrant activity of the transcription factors NF-κB and FOXO1 (Forkhead box protein O1) (106) during obesity or inflammatory conditions. Therefore, IL1β has physiological roles in glucose metabolism with deleterious consequences after chronic exposure to high concentrations.

Macrophages play a crucial role in liver inflammation. Resident hepatic macrophages undergo activation and thereby alter inflammatory pathways in obesity conditions (107). In addition, there is a pronounced increase in macrophage (and other immune cell) infiltration in the liver. This leads to production of inflammatory cytokines that generate insulin resistance in hepatocytes and drive T2D-related diseases such as non-alcoholic fatty liver disease (NAFLD) and non-alcoholic steatohepatitis (NASH) (108, 109). Nevertheless, a recent study in mice and humans with obesity could not confirm that obesity *per se* induces a pro-inflammatory phenotypic switch in liver macrophages (110), although their depletion prevents diet-induced insulin resistance (111). Interestingly, liver macrophages were shown to contribute to the development of insulin resistance independent of production of inflammatory factors. Rather, liver macrophages were shown to produce insulin-like growth factor–binding protein 7 (IGFBP7), which directly altered insulin receptor signaling in the liver (110).

Inflammation is a biological response of the immune system that can be triggered by exposure to pathogens, damaged cells and toxic compounds. The reaction can be acute or chronic, potentially leading to damage in several tissues. In particular, bacterial components such as lipopolysaccharide (LPS), a component of the cell wall of Gram-negative bacteria, have been proposed as a source for metabolic inflammation since LPS was found to be increased in the circulation of people with diabetes (112). LPS was postulated to enter the circulation via chylomicrons or via increased intestinal permeability (113) where it induces an inflammatory response in systemic sites. In mice, chronic LPS infusion perturbed glucose tolerance by inducing hepatic insulin resistance and hampering glucose-stimulated insulin secretion (114). In humans, associations between LPS concentrations and several aspects of the metabolic syndrome were also noted (112, 115).

Two phenomena have been noted: “metabolic endotoxaemia” (116, 117) and “postprandial inflammation” (118). The former describes an inflammatory response to increased systemic levels of LPS due to a “leaky gut” (114). The latter defines the increase in circulating endotoxins and other inflammatory markers after a meal, particularly meals rich in fat (119–121). Interestingly, LPS and long-chain saturated fatty acids, such as palmitate, act synergistically in activating inflammatory signaling in macrophages (122), highlighting a link between a westernized diet rich in saturated fatty acids (123) and postprandial inflammation.

From the present data, it is clear that a dysregulated immune system is strongly associated with obesity and T2D. Moreover, several inflammatory components directly alter glucose tolerance and insulin sensitivity, which provides evidence for a causal role of inflammation in these pathologies. The intertwined relation between metabolic disorders and low-grade inflammation has been coined “metainflammation.” This illustrates that, in contrast to an instrumental acute inflammatory response to pathogens and tissue damage, chronic low-grade inflammation as observed in obesity and T2D has detrimental consequences for human metabolism.

## The Intestinal Microbiota in Metabolic Diseases

Within the human intestine, a complex mutualistic relation exists between trillions of microorganisms, collectively known as the gut microbiota, and the host (124). While much focus is on the bacterial component of this community, recent studies highlight the importance of other microorganisms such as bacteriophages (125, 126) and fungi (127, 128) in human health. It is beyond the scope of this review to cover the role of other members of the microbial community in human metabolism. We will here focus on the bacterial component that we will refer to as microbiota. The gut microbiota is critical for human health (129–131). It plays a key role in digestion, production of metabolites with the potential to alter human metabolism (e.g., short chain fatty acids) and development of the immune system. The immunomodulatory properties of the gut microbiota are of particular interest in the context of metainflammation as observed in obesity and T2D (132).

Germ-free mice housed in aseptic conditions, have an underdeveloped immune system. These mice typically have impaired development of gut-associated lymphoid tissues (GALT), decreased serum immunoglobulin levels, smaller spleens and mesenteric lymph nodes with low levels of tissue resident macrophages (133). In line, the mice have an insufficient immune response to pathogens (134, 135). Introducing bacteria into germ-free mice restores immune system formation and functioning which exemplifies the critical and dynamic relation between host immunity and the gut microbiota (136). These findings have not only been related to presence of bacteria but also to the microbial metabolites such as SCFA (137) or components such as commensal DNA containing CpG (unmethylated cytosine phosphate guanosine dinucleotides) motifs (138) and polysaccharide A (PSA) (139). The development of a symbiotic relationship during early years may determine the development of several diseases (140).

Importantly, germ-free mice are resistant to diet-induced obesity (141, 142). Introducing a single bacterium that has been associated with obesity (*Enterobacter cloacae*) into germ-free mice led to weight gain, a disturbed glucose tolerance, higher systemic lipopolysaccharide binding protein (LBP) concentrations and lower adiponectin levels (143). Similarly, germ-free mice that received the microbiota from obese donors gained more energy from the diet compared to germ-free mice that received the microbiota of lean donors (144, 145). These studies were the first to show that an obese phenotype can be transferred via the gut microbiota and hence indicate causality.

**Figure 3:**
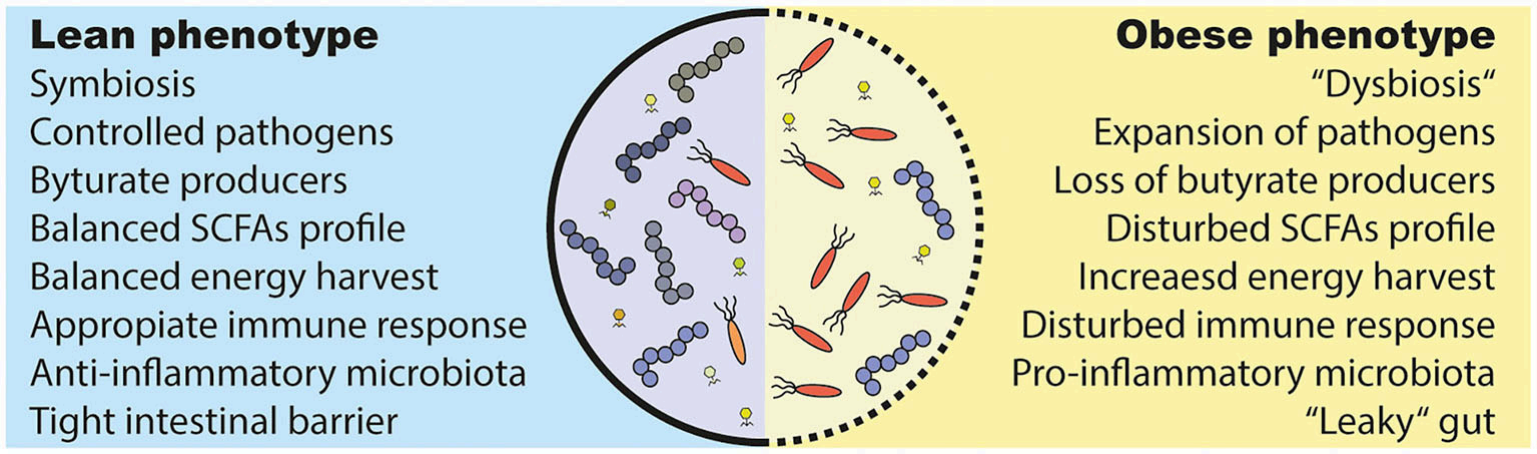
Alterations in the obese and diabetic gut microbiota. (1) Under healthy conditions (lean), the gut microbiota lives in symbiosis and provides the host with several beneficial functions. For example, it produces short chain fatty acids (SCFAs) that are used as an energy source and have effects on several host tissues. However, several bacteria are able to induce an inflammatory response and can even breach the intestinal barrier, which has to be prevented by a proper immune response. (2) The gut microbiota in metabolic diseases is often described as “dysbiotic,” meaning that there is an expansion of normally underrepresented bacteria (in particular opportunistic pathogens) and a lower diversity. A disturbed intestinal immune response and a westernized diet is discussed as causes. Further, a westernized diet induces a “leakiness” of the gut. Parts of (opportunistic) bacteria are able to cross the intestinal barrier and induce a pro-inflammatory response in the host. Lastly, people with obesity show an increased energy harvest by the gut microbiota and a different SCFAs profile as lean people, which might have deleterious consequences for the host health.

Although a definition of a “healthy gut microbiota” is generally lacking, a plethora of disease conditions are associated with a microbiota composition that is different from a healthy control group (124, 146). The most preferred term in this context is microbial “dysbiosis,” which can describe a bloom of pathobionts, loss of commensals or/and loss of diversity (147). However, the term falls out of favor due to lack of a clear definition. An altered microbiota is present in people with obesity (144, 148) and T2D (6, 149) (Figure 3). It is generally characterized by expansion of usually underrepresented microorganisms (often opportunistic pathogens) (6, 143) and lower phylogenetic alpha diversity (8, 150, 151). Diet (152) and antibiotic use (153) have been identified as drivers of these changes.

The obesity-associated microbiota has been shown to extract more energy from the diet compared to lean controls, proposing a role in the development of excessive energy stores (144, 145, 154). Further, an enrichment of pro-inflammatory bacteria (e.g., *Escherichia coli*) at the expense of anti-inflammatory bacteria (e.g., *Fecalibacterium prausnitzii*) in T2D has been reported several times (6, 7, 149). An increase in typical pro-inflammatory Gram negative bacteria might be a plausible source for the metainflammation seen in metabolic diseases (155, 156). However, this is only confirmed in animal studies and in associative nature in humans.

A reduced number of anti-inflammatory bacteria such as *F. prausnitzii* has been related to a disturbed SCFA production of the gut microbiota (6). SCFAs, which result from microbial degradation of fibers, have several beneficial effects on host metabolism (157). Surprisingly in obesity, an increased SCFA production has been observed (158, 159), which was proposed to be responsible for higher energy extraction (160) and was dependent on the diet (161). It is estimated that 5–10% of our daily energy is provided by fermentation processes by the gut microbiota (162). However, in diabetes a reduced SCFA production or uptake, in particular the anti-inflammatory acting butyrate (163), has been suggested (6). However, strong evidence for this concept is still lacking. A recent study found a causal relation between a host genetic-driven increase in butyrate production which was associated with an improved insulin response. *Vice versa*, abnormalities in the production or absorption of propionate were related to increased risk of developing T2D (164). Absorption of intestinal SCFAs is very efficient, leaving only 5–10% in feces (165). Large amounts of the intestinal butyrate is directly used by the colonic enterocytes as an energy source (166, 167). However, it is difficult to measure real turnover and concentrations of SCFAs due to rapid utilization by intestinal cells and microorganism. Thus, although SCFAs are involved in host metabolism, the mechanism and extent are still elusive.

In summary, changes of the gut microbial composition and a lower microbial diversity in obese subjects has been associated with higher inflammatory tone (8, 168, 169). This implicates a role for the gut microbiota in the low-grade inflammation observed in people with metabolic syndrome (170, 171).

## Gut Barrier Function and Metabolic Inflammation

A proper gut barrier function is critical to prevent bacterial infiltration from the gut into the circulation and periphery. Many pathologies, including inflammatory bowel disease (172), liver diseases (173), and metabolic syndrome (114), show signs of a disturbed gut barrier function leading to bacterial translocation (174). A “leaky” gut can facilitate translocation of bacterial components form the intestine into the periphery (174). The following part summarizes evidence of bacterial leakage in metabolic diseases.

### Intestinal Barrier Integrity

**Figure 4:**
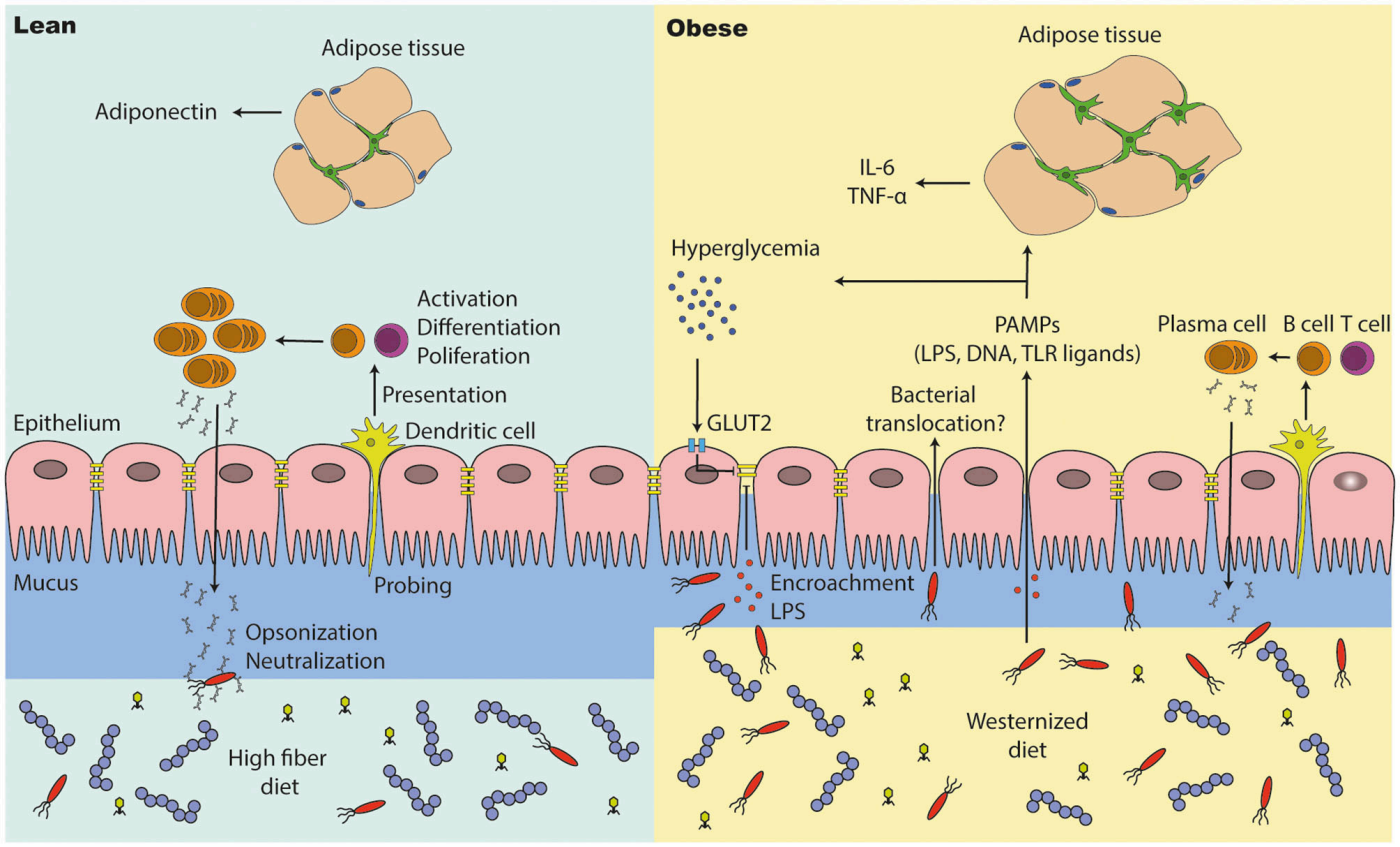
The intestinal barrier is disturbed in people with obesity and diabetes. (1) A high fiber diet supports intestinal barrier function by improving intestinal tight junction expression and immune cell function. Antigen presenting cells (e.g., dendritic cells) are probing the intestinal environment, present the antigens to T and B cells, which may lead into immune tolerance or an inflammatory response (cytokine and antibody expression). (2) The intestinal integrity is affected in people with metabolic syndrome. They have a thinner mucus layer, which leads to penetration of opportunistic bacteria; lower levels of IgA positive B cells and a lower IgA secretion, which may end into microbial alterations (outgrowth of opportunistic pathogens). A westernized diet decreases intestinal tight junction expression, which results into translocation of bacteria and pathogen associated molecular patterns (PAMPs). High glucose levels (hyperglycemia) reduces tight junction expression via GLUT2, promoting bacterial translocation in people with diabetes. PAMPs in the periphery induce inflammation in several other tissues such as the adipose tissue, where macrophages proliferate and accumulate. In particular, adipose tissue macrophages are responsible for low grade inflammation (high pro-inflammatory cytokine levels and less anti-inflammatory cytokines).

One of the first papers implicating a link between gut barrier function, insulin resistance and increased levels of circulating levels of LPS were derived from a study in obese mice (114) (Figure 4). Antibiotic treatment reduced intestinal and systemic LPS along with an improved glucose tolerance, underscoring the gut microbiota as the endotoxin source (155). Mechanistically, these findings were related to high-fat diet (HFD) induced reduction in expression of tight junction genes, which was linked to increased intestinal permeability (155, 175). Tight junctions are protein complexes that prevent leakage of various compounds along paracellular spaces (175). LPS directly increased intestinal permeability *in vitro* and *in vivo* in mice, suggesting a link between increased intestinal LPS and the gut tight junction expression (176).

Interestingly, hyperglycemia directly drives intestinal barrier permeability though Glut2-dependent reprogramming of intestinal epithelial cells (177). Loss of gut integrity increased influx of bacterial products in the periphery of mice and potentially humans, proposing an interplay between inflammation and a disturbed glucose tolerance. Further, systemic toll like receptor (TLR) ligands were positively correlated with the long-term glucose marker HbA1c in humans (177). Although metabolic endotoxemia in obesity and T2D becomes more evident, human studies showing a higher intestinal permeability and disturbed tight junction expression are still lacking.

Several groups tried to identify “translocating” bacteria in peripheral organs and tissues by measuring or sequencing the 16s ribosomal RNA of bacteria. First, more bacterial DNA was found in the circulation of diabetic subjects and obese mice compared to healthy controls, with predominantly Proteobacteria identified (178). Secondly, DNA from Gram-negative bacteria was detected in adipose tissue of obese women and correlated with a decrease in adiponectin levels, suggesting a link between bacterial translocation and activation of inflammatory pathways in (adipose) tissue (179). Further, an increased adherence of pro-inflammatory bacteria to the epithelium might indicate that bacteria are passing the epithelium (178). However, the concept or bacterial translocation is still very controversial due to technical limitations to detect small amounts of bacteria in systemic tissue sides.

### Immunoglobulins

Immunoglobulins control the gut microbiota and prevent bacterial invasion by binding to microorganisms directly to block the attachment to the host. In addition, immunoglobulins binding marks (so called “opsonization”) bacteria for phagocytosis and antigen presentation to dendritic cells. Moreover, immunoglobulins neutralize microbial toxins (180). A proper induction in childhood is essential to build up an immune tolerance against gut bacteria and to prevent microbial changes (181, 182). Particularly, secretory IgA are pivotal players in shaping gut microbiota composition, both in mice and humans, and defective IgA secretion has been shown to shift microbial communities (183–185). An altered intestinal immunoglobulin response has been noted for several diseases including undernutrition (186), inflammatory bowel disease (187), and obesity (188).

A recent study reported fewer IgA+ immune cells and less IgA secretion in obese mice (188). Further, IgA deficient obese mice had impaired glucose tolerance, higher macrophage content in adipose tissue and more systemic endotoxins. Antibiotic treatment improved glucose tolerance, suggesting the involvement of the gut microbiota in this phenotype. The anti-diabetic drug metformin improved IgA response in obese mice and bariatric surgery increases fecal IgA in human subjects (188). Further, a higher flagellin expression of motile bacteria and a disturbed antibody response against flagellins leading to bacterial encroachment on the intestine has been discussed in obesity and inflammatory bowel diseases (189).

Immunoglobulins are produced by B cells, which had impaired function in T2D (190). B cells accumulated in visceral adipose tissue of obese mice (191) and produced more pro-inflammatory cytokines compared to B cells in adipose tissue of lean controls (192). B cell depletion in obese mice decreased inflammation and improved glucose tolerance (192), suggesting that disturbed B cell function in obesity is linked to inflammation and glucose metabolism.

T cells, particularly follicular helper T cells, are important players in the induction of a proper immune and antibody response (193, 194). Depletion of their function led to a disturbed intestinal IgA activity and the development of metabolic syndrome with age. IgA inappropriately targeted Clostridia species and allowed for the outgrowth of Desulfovibrio. The former suppresses and the latter enhances host lipid absorption via CD36 modulation (194), which plays essential role in fatty acid uptake (195). This study gave insight in the development of microbial alterations, which are present in metabolic diseases, via IgA. It is clear that immunoglobulins play a major role in gut homeostasis (196), but only a few recent studies show the involvement of immunoglobulins in the disease progression of metabolic diseases.

Bacterial translocation, as discussed above, might be an interesting concept to explain inflammatory changes in obese and diabetic tissues that leads into insulin activity loss (beta-cell dysfunction and insulin resistance). However, bacterial translocation as a concept is still very controversial due to lack of strong evidence in humans. There currently is a trend to look into the role of bacterial metabolites as causative in the development of metabolic diseases rather than presence or absence of bacterial strains. In the following part, we will discuss evidence of metabolites involved in metabolic disease progression.

## Mechanistic Insights in Microbiota-Induced Metabolic Inflammation

Low grade chronic inflammation in obesity and T2D has been studied for more than 25 years (60), with several pathways to a large extend disentangled (197). In contrast, observations on changes in the gut microbiota of obese and diabetic people were revealed no longer than 15 years ago (6, 144, 198). Particularly a westernized lifestyle is known to change not only the immunemetabolism, but also the gut microbiota (199). The connection between gut microbial changes and metainflammation in metabolic diseases was established even more recently. The following part addresses mechanistic insights from rodent studies and the few causal relationships found in humans.

### Pattern Recognition Receptors

**Figure 5:**
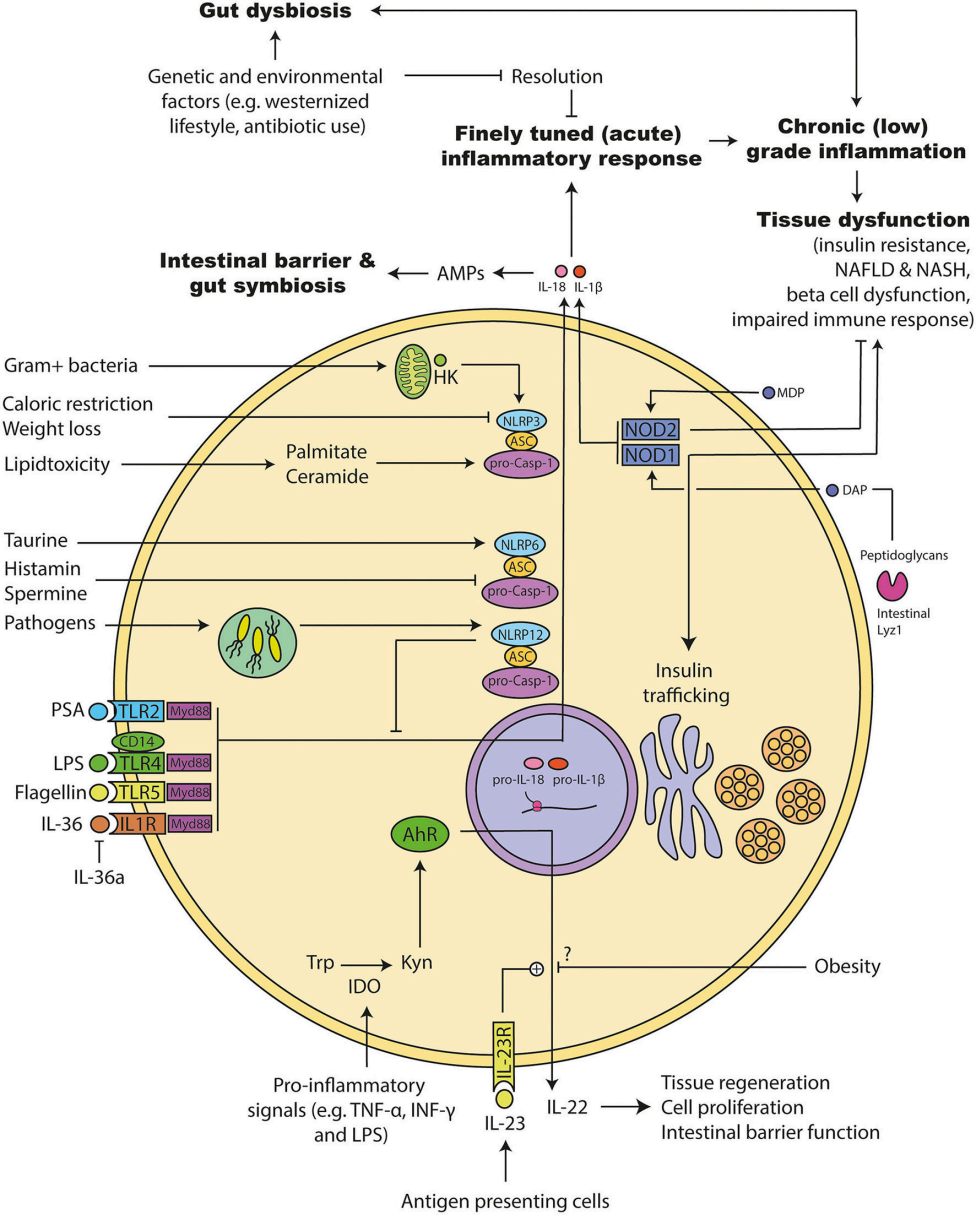
Molecular mechanism involved in microbiota promoted metainflammation. (1) The acute inflammation has to be resolved to avoid chronic inflammation that can induce tissue damage. Genetic and environmental factors can disturb this system leading to a chronic (low-grade) inflammation. Several of following pathways are disturbed during obesity and diabetes: (2) Tolle like receptors (TLRs) and their adapter molecules are important for recognizing bacterial components. Activation triggers different inflammasomes to initiate an inflammatory response. Similarly, interleukin (IL) 36 leads to the activation of the inflammasomes and a pro-inflammatory response that can be inhibited by the endogenous IL-36 antagonist. (3) Inflammasomes consists of different proteins: NACHT, LRR, and PYD domains-containing protein (NLRP), Apoptosis-associated speck like protein containing a caspase recruitment domain (ASC), and pro-caspase. Upon activation they can mature IL-1β and IL-18. NLRP12 has dual roles: It acts pro-inflammatory response via maturation of IL-1β and anti-inflammatory by inhibiting down-stream signals of several TLRs. NLRP6 is important for the maturation of IL-18 and antimicrobial protein expression in the intestine. Its activity can be increased by the microbial metabolites taurine and decreased by Spermidine as well as Histamine. It is important for maintaining a gut symbiosis and intestinal barrier function. NLRP3 activity can be increased during lipid accumulation. Further, hexokinases can detect intracellular particles of Gram positive bacteria and activate NLRP3, which leads to the maturation of the pro-inflammatory acting IL-1β. (4) Pro-inflammatory signals can increase the intracellular enzyme indoleamine 2,3-dioxygenase (IDO), which in turn metabolizes tryptophan to kynurenine. Kynurenine can activate the transcription factor Aryl hydrocarbon receptor (AhR), which induces the release of IL-22. IL-22 is important for the intestinal barrier function, which can be promoted via IL-23. Obesity interferes with that response, but the exact mechanism is not clear. (5) Nucleotide-binding oligomerization domain-containing protein (NOD) 1 can be activated by bacterial diaminopimelic acid (DAP). DAP can be cleaved by intestinal Lyzozyme (lyz) 1 enzymes from bacterial peptidoglycans. NOD1 has dual roles: It induces insulin resistance and insulin trafficking in beta cells. NOD2 can be activated by bacterial muramyl dipeptide (MDP). NOD2 inhibits the development of insulin resistance.

The intestinal epithelium is the first line of defense against intestinal microorganism (200). A tight intestinal wall with a properly working immune system is necessary to avoid invasion. Pattern recognition receptors (PRR) are expressed on most cells of the innate immune system. PPRs recognize microbial components and hence are crucial parts of the immune system. They include the widely-studied membrane-bound TLR superfamily. These receptors are mainly expressed on epithelial cells and cells of the innate immune system (201). TLRs detect pathogen-associated molecular patterns (PAMPS) such as LPS or flagellin and facilitate an inflammatory response (202). Acute inflammation is important to clear of infected, abnormal or damaged tissue. The process needs to be resolved to avoid unnecessary damage on healthy tissue. Usually this resolution is disturbed in conditions of chronic inflammation (203) (Figure 5).

TLRs are a crucial tool of our immune system to detect invading microorganism. Several TLRs have been associated with the development of the metabolic syndrome, in particular TLR2 and TLR4 (204). TLR4 recognizes several ligands, but most predominantly LPS (205). Monocytes from people with T2D showed higher expression of TLR4, along with higher cytokine and LPS levels (206). Interestingly, insulin infusion for several hours is able to suppress the expression of TLRs on monocytes from people with T2D (207), suggesting that insulin resistance promotes higher expression of TLRs (and thereby inflammation). Further, TLR4 has been suggested to play a role in beta cell failure during diet-induced obesity in mice, as HFD-fed TLR4 knock out mice exhibit preserved insulin secretory function, lower inflammatory markers and no macrophage infiltration in pancreatic islets (208). Further, a combined knockout of TLR4 and TLR2 in mice increased proliferation of beta cells, but not glucagon-producing alpha cells, and improved glucose tolerance in obese mice (209). Interestingly, absence of CD14, a co-receptor of TLR4, protected mice from most of HFD and LPS induced metabolic deteriorations (114). Thereby, TLR4 might be an interesting treatment target and an important mediator of insulin resistance, metainflammation and beta cell failure.

TLR5 has been associated with the development of metabolic diseases (210). TLR5 is expressed on several cell types and recognizes mainly flagellins, a protein involved in the motility of bacteria (211). Knock out of TLR5 in mice led to the development of metabolic syndrome, which correlated with changes in the gut microbiota. Transfer of feces from TLR5 knockout mice to wild type mice conferred many features of the metabolic syndrome (210). Importantly, follow-up studies suggest that the observed metabolic changes were likely due to housing techniques rather than the genetic profile (212). TLR5 knock out in intestinal epithelial cells induced low grade inflammation and metabolic syndrome, which was reversed by antibiotics, implying a connection with the gut microbiota (213). TLR5 is likely involved in the development of a metainflammation.

Myeloid differentiation factor 88 (Myd88) is a downstream target of most TLR- and IL-1 receptor-mediated signaling pathways and a central player in innate immune signaling (214). Myd88 knock out in mice induced metabolic syndrome with an increase in bacterial translocation (178). Further, hepatocyte specific deletion of MyD88 predisposed mice to glucose intolerance, inflammation, hepatic insulin resistance and induced gut microbiota alterations (215). In contrast, inducible deletion of Myd88 in intestinal epithelial cells partially protected against diet-induced obesity, diabetes and inflammation. The protective phenotype was transferred by transplanting feces from intestinal Myd88 knock out mice into germ-free mice. Targeting MyD88 (inducible knock out) after onset of obesity reduced fat mass and inflammation, suggesting a link between microbiota and MyD88 in the development of obesity (216). A finely tuned MyD88 activation therefore is crucial for the homeostasis of several tissues and is involved in metabolic diseases.

Similar to TLRs, nucleotide-binding oligomerization domain-containing protein 1 (NOD1) and NOD2 recognize PAMPs inside of the cell. NOD1 and NOD2 are cytosolic receptors that respond to bacterial peptidoglycans (217) and have been associated with the development of insulin resistance (218, 219). Interestingly, NOD1 and NOD2 double knock out protected mice from inflammation and peripheral insulin intolerance. Direct NOD1 activation led to insulin resistance (218) and gut microbial derived NOD1 ligands promoted insulin trafficking in beta cells (220). The latter is most likely a compensatory mechanism after the development of an insulin resistance. On the contrary, NOD2 signaling is protective against T2D. Defective NOD2 sensing promotes diet-induced inflammation, microbial changes and insulin resistance (221). NOD2 activation via bacterial cell wall-derived muramyl dipeptide (MDP) improved insulin resistance (219). From the present data it seems that NOD2 signaling is beneficial whereas NOD1 is deleterious for insulin sensitivity and beta cell function.

### Inflammasomes

Inflammasomes are a central component of the innate immune response and have been implicated in several metabolic diseases (222). Inflammasomes are intracellular multimeric complexes composed by NLRs (NOD-like receptors), ASC (Apoptosis-associated speck-like protein containing a CARD), and pro-caspase-1. They are formed in response to PAMPs and DAMPs (damage associated molecular patterns) and control the activation and secretion of IL-1β and IL-18 (223). Thereby, they act as intracellular sentinels of inflammatory and metabolic cellular disturbances. Knocking out of several NLRs in mice led to changes in the gut microbiota (224–229) and aggravation of diet-induced metabolic syndrome (229).

NLRP3 (NOD-like receptor family, pyrin domain-containing 3) is one of the most studied NLRs. It responds to a wide range of infectious and endogenous molecules, such as several bacterial cell wall components (222, 230) and saturated fatty acids (97). However, none of these molecules seem to directly interact with NLRP3. Common cellular signals might activate NLRP3 (231). That priming process leads to the activation of caspase-1 and subsequent IL-1β secretion (232). Several studies linked increased NLRP3 expression in adipose tissue and monocytes to obesity (233) and T2D (234), respectively. Interestingly, calorie restriction in obese and diabetic subjects reduced activation of NLRP3 in adipose tissue and coincided with reduced inflammation and improved insulin sensitivity (232).

Further, there is a direct connection between cell metabolism and inflammation. Hexokinases, enzymes that are mainly involved in glucose metabolism, were able to detect bacterial peptidoglycans and thereby activated NLRP3 (230). Several studies disclosed that an ablation of NLRP3 or of the inflammasome components ASC and caspase-1 in mice prevented obesity-induced inflammation in fat depots and liver, ameliorated insulin signaling, increased energy expenditure and prevented liver steatosis as well as pancreatic damage (225, 232). Mechanistically, NLRP3 inflammasome participated in metainflammation and lipotoxicity by sensing intracellular rise of the sphingosine ceramide (232), the saturated fatty acid palmitate (97) and reactive oxygen species (235). Therefore, NLRP3 is a major component in the inflammatory processes deranged in metabolic diseases.

Further, ablation of ASC, a common adapter molecule to all inflammasomes, on a *db/db* (leptin-deficient mice) background resulted in increased obesity and loss of glycemic control under high fat diet conditions. Moreover, this phenotype was transmissible to wild type mice by co-housing and abrogated by antibiotic treatment pointing to an essential role of gut microbiota in the phenotype. In the same paper, the authors found that deficiency in components of the inflammasome (caspase-1, ASC, NLRP3, or NLRP6) aggravated the progression from NAFLD to NASH and disease severity, and that this phenotype was transmissible to wild type mice by co-housing. These effects were attributed to a different cologenic microbiota in inflammasome deficient mice that increased the amount of TLR4 and TLR9 bacterial agonists into the portal circulation, driving hepatic TNF induction and, hence, NASH (225). However, the authors used unrelated wild-type mice instead of littermate controls. Two later studies highlight the importance of using proper controls, namely related littermates from the knock out mice, to exclude the influence of housing conditions and other genetic variations in the littermates (236, 237). Nevertheless, these findings suggest a role of inflammasomes and their components in several comorbidities of obesity.

Interestingly, Levy et al. provide evidence of a direct link between commensal-derived metabolites and NLRP6 inflammasome activity (227). The authors found that histamine and spermine inhibited NLRP6-dependent IL-18 production, while taurine enhanced it and consequentially stimulated the IL-18 driven production of antimicrobial peptides in the intestinal epithelium. However, a two later papers disputed the impact of NLRP6 and ACS-associated inflammasome in shaping the microbiota composition using littermate-controlled experimental design in two geographically separated vivarium to compare the phylogenetic composition of wild type, NLRP6^−/−^, ASC^−/−^ mice microbiota and minimize non-genetic confounders (236, 237). Therefore, NLRP6 and its components are involved in metainflammation via the gut microbiota, but the exact mechanism is still elusive.

The NLRP12 inflammasome has more recently been implicated in the development of metabolic diseases. Interestingly, it was first though to induce a pro-inflammatory response, but recently a dual role became evident (238). It has been shown to dampen and resolve inflammation, in particular in the intestine, in a microbiota dependent manner (228) and by promoting the growth of beneficial bacteria (228). NLRP12 expression in adipose tissue was reduced in obese compared to lean subjects. In mice, deletion of NLRP12 increased weight gain, adipose tissue deposition, blood glucose and pro-inflammatory macrophage expansion. Interestingly, NLRP12 knock out in mice induced gut microbiota alterations with decreased number of SCFA producing bacteria. Depletion of the microbiota, germ-free condition or co-housing with wild type mice was sufficient to restrain inflammation, obesity, and insulin tolerance in knock out mice, highlighting a direct link of the gut microbiota in obesity and inflammation (229). Overall, these findings form a strong link between microbiota, inflammasomes, and metabolic diseases.

### Cytokines

Several cytokines are associated with the development of the metabolic syndrome (as discussed above), but only a few studies found direct links between cytokines and the gut microbiota. IL-22 is essential for maintaining an antimicrobial response and intestinal barrier function (239). Its production is induced by intestinal type 3 innate lymphoid cells (ILC3) and influenced by bacterial tryptophan metabolism (227, 240, 241). Induction of IL-22 was impaired in obese mice, in particular during infection with *Citrobacter rodentium* (242), suggesting an impaired immune response against pro-inflammatory bacteria. Mice lacking the IL-22 receptor on a high fat diet are prone to develop metabolic syndrome and administration of IL-22 in obese mouse models reversed several symptoms such as hyperglycemia and insulin resistance (242, 243).

Protective effects of IL-22 on pancreatic islets may explain these findings. Administration of IL-22 reduced ER stress and inflammation with a restored glucose tolerance in mice (244). Some bacterial metabolites such as butyrate were able to induce IL-22 secretion from pancreatic innate lymphoid cells, which improved beta cell proliferation and inflammation (245). Next, inactivation of IL-22 (and IL-23) signaling in mice deteriorates intestinal barrier, microbial alterations and expansion of pathogenic bacteria causing systemic increase in LPS and Trimethylamine N-oxide (TMAO), showing direct connection of those particular cytokines, the gut microbiota and obesity related comorbidities such as atherosclerosis (246). On the other hand, T cell derived IL-22 amplified IL-1β driving inflammation in human adipose tissue (190), suggesting either a tissue specific activity of IL-22 or a disturbed action in metabolic syndrome.

Further mechanistic insights of the IL-22 signaling were given in a recent studies, where indoleamine 2,3-dioxygenase 1 (IDO), an enzyme that is present in many immune cells and activated during inflammation, was linked to the gut microbiota and obesity (247). IDO metabolizes tryptophan along the kynurenine pathway, which is a potent AhR ligand and shown to disturb an anti-tumor response (248). Interestingly, it becomes more evident that the tryptophan metabolism plays an important role in metainflammation (249) and energy homeostasis (250). Although IDO activity is important for a proper regulatory T cell function (251), a high enzymatic activation is associated with cardiovascular complications and inflammation (252). The enzyme activity was shown to be increased in obesity (247). Deletion or inhibition of the enzyme improved insulin sensitivity, improves gut barrier and chronic inflammation. Neutralization of IL-22 abrogated the protective effects of IDO deletion, highlighting the beneficial roles of IL-22 in intestinal health and glucose tolerance (247). More studies are needed to elucidate the dysregulation of the tryptophan metabolism and its metabolites in metainflammation.

ILC3s are an important source of IL-22 in the intestine, which is regulated by the adaptive immune system (CD4+ T cells) (253). The lack of CD4+ T cells led to an upregulated activity of intestinal ILCs that disturbed the host-microbe interaction and reduced intestinal lipid metabolism (253). Further, elevated levels of IL-23 and IL-22 in young mice decreased the production of pancreatic enzymes, explaining the disturbed lipid metabolism to some extend (254). However, inactivation of both cytokines led to deterioration of the intestinal barrier, dysbiosis and a pro-atherogenic environment (increase in LPS and TMAO) (246). These findings highlight a fine tuned interplay between various immune cell types and mediators. Generalizing them as pro- or anti-inflammatory as well as beneficial or deleterious is in most cases not possible.

IL-23 has mainly pro-inflammatory activities (255), which is mostly secreted from activated macrophages and dendritic cells (DCs) located in peripheral tissues (256). Further, it induces the production and secretion of IFN-γ from various cell types, upon recognition of bacterial, viral and fungal components (257). Further, IL-23 is important for the development of T helper 17 cells (Th17) (258), which are in turn critical players in the homeostasis within the gut (259). Interestingly, obese women had higher circulating levels of IL-23 compared to healthy controls (260), supporting the assumption of a pro-inflammatory environment in metabolic diseases. Surprisingly, mice lacking IL-23 on a high fat diet, gained more weight and were more glucose intolerant than the controls (261). These mice also exhibit an increased gut permeability and bacterial translocation compared to wild type mice, underlining the important of IL-23 in the gut homeostasis.

Recently, IL-36 has been discovered and associated with obesity. IL-36 belongs to the IL-1 receptor family (262) and promotes the resolution of intestinal damage (263). IL-36 expression was increased in obese patients and negatively correlated with high blood glucose levels (264). Mice lacking an endogenous inhibitor for the IL-36 receptor had better glucose tolerance and insulin sensitivity compared to controls. Lack of IL-36 inhibition increased the abundance of *Akkermansia muciniphila*, mucin formation and intestinal integrity (264). However, this is the sole study indicating a role of IL-36 in metabolic diseases.

Lastly, typical anti-inflammatory cytokines (as discussed above) have essential roles in maintaining the intestinal homeostasis. For example, IL-10 is important to dampen an inflammatory response against intestinal bacteria (265). Particularly, microbiota derived SCFAs can stimulate the protection of IL-10 (266). Knock out of IL-10 induces colitis in mice, which is dependent on the microbiota since germ-free mice do not develop colitis (267). However, studies in metabolic diseases focuses mostly on the circulation than the intestine of obese humans or rodents.

Although several cytokines have been associated with metainflammation in obesity and T2D, only a few show direct connection to the gut microbiota such as IL-22, IL-23, and IL-36. However, several others have been discussed in the context of intestinal health and immune function (268), which have not been associated with metabolic inflammation yet. It is plausible that more cytokines are involved in all three processes. The ongoing discovery of new bacterial metabolites will shed more light into the complex pathways.

## Microbiota-Produced Metabolites and Host Inflammation

The hypothesis that bacteria physically translocate to tissues, where they may invoke an inflammatory response has generated mixed results in literature as described above. A more recent line of reasoning, which relates gut microbiota to inflammation and cardiometabolic disease, includes the production of gut-derived metabolites that enter the systemic circulation to induce several effects. These metabolites have been linked to specific intestinal microbiota. Several of these metabolites have received much attention in recent years (269). Recently, metabolites such as imidazole propionate were discovered and shown to be involved in insulin resistance (270), however only for a few bacterial metabolites a direct connection between metabolism, immune system, and gut microbiota has been made.

### Fibers and Short Chain Fatty Acids

**Figure 6:**
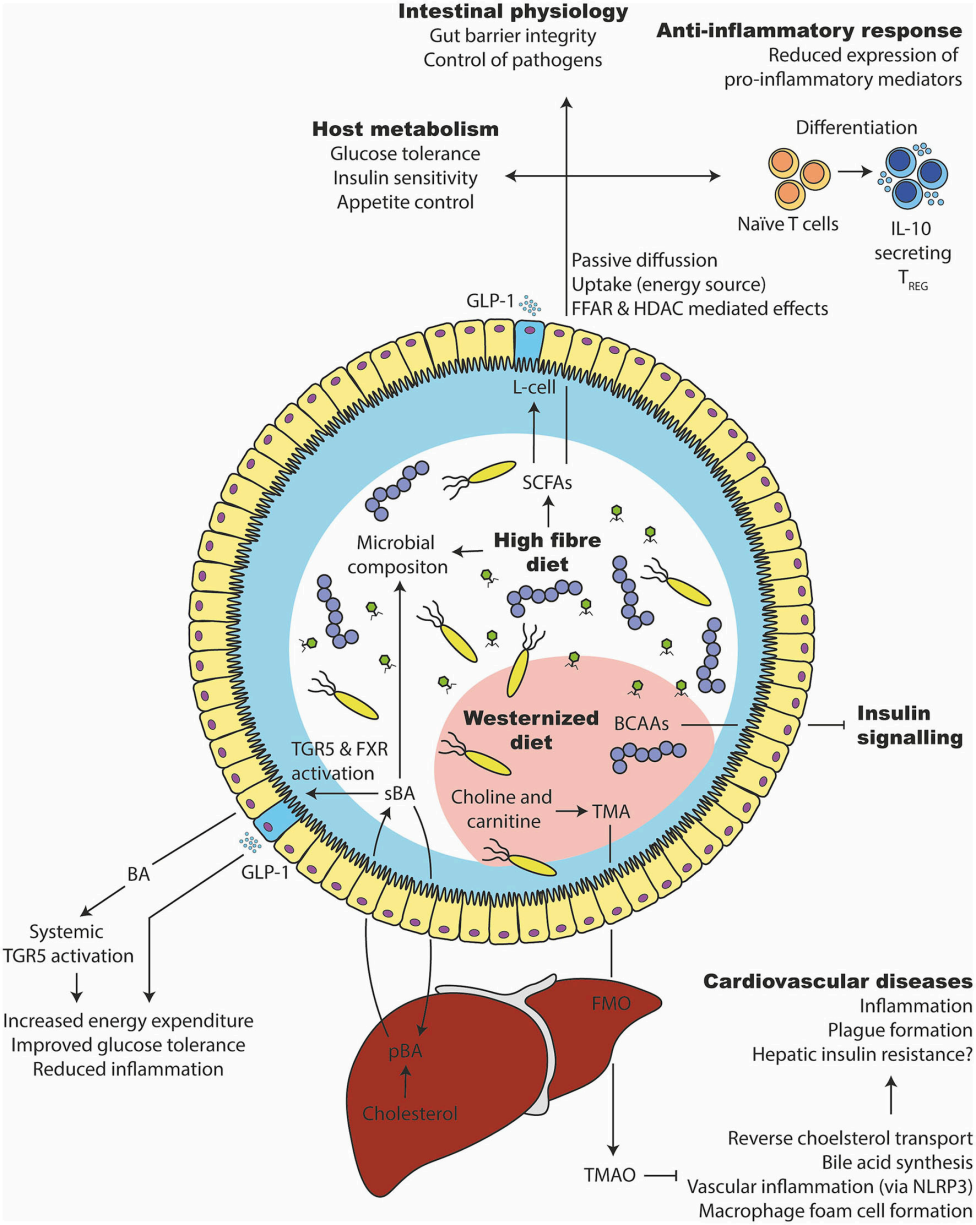
Microbial metabolites that affect glucose tolerance and inflammation. (1) A high fiber consumption has several beneficial effects on the gut microbiota and host health. They are degraded by the gut microbiota in short chain fatty acids such as butyrate, propionate, and acetate. SCFAs can be taken up by the enterocytes, used as an energy source or bound to free fatty acid receptors to stimulate varies responses (e.g., GLP-1 release from intestinal L-cells). Intracellularly, it can stimulate epigenetic changes via histone deacetylase (HDAC). It supports the expansion of beneficial bacteria and keeps opportunistic pathogens in control, improves glucose and appetite control, supports intestinal barrier integrity and induces an anti-inflammatory immune response in intestinal as well as systemic tissue sides. (2) Primary bile acids (pBA) are produced in the liver from cholesterol and secreted into the intestine via the gall bladder. There, they can change the gut microbiota and are transformed by bacteria to secondary bile acids (sBA). Bile acids can activate intestinal and systemic TGR5 as well as FXR, which increases the energy expenditure, lower inflammation and improves glucose tolerance. (3) A westernized diet, which is usually rich in saturated lipids, can disturb the branched chain amino acid (BCAA) catabolism of the host, which in turn inhibits the insulin signaling. (4) Further, a westernized diet is commonly rich in choline and carnitine, which the gut microbiota can metabolize to trimethylamine (TMA). After intestinal uptake, the liver transforms TMA into Trimethylamine N-oxide (TMAO) via flavin-containing monooxygenase (FMO). TMAO inhibits bile acid synthesis, reverse cholesterol transport (RCT), induce macrophage foam cell formation and inflammation via NLRP3. That in turn leads to cardiovascular complications.

The most widely studied metabolites include the short-chain fatty acids (SCFA) acetate, butyrate and propionate, generated by gut microbiota which ferment indigestible dietary components such as complex carbohydrates (fibers) (165). In that regard, high fiber intake was associated with a protection from several metabolic diseases such as T2D (271). Further, fiber intake promoted the expansion of SCFAs producing bacteria and improved glucose tolerance (partly via GLP-1) in people with T2D (272) (Figure 6). Inulin (high fermentable fiber) supplementation increased insulin sensitivity in human subjects. Inulin propionate ester improved insulin sensitivity as well as reduced IL-8 (273), suggesting beneficial effects on inflammatory parameters. Furthermore, high fiber intake promoted gut barrier integrity in mice, which was associated with the SCFAs production (274).

SCFAs have been linked to a several metabolic processes including induction of appetite regulation (275, 276) and improving insulin resistance in muscle and adipose tissue (165, 277, 278). However, not all SCFAs seem to have beneficial effects. Acetate was shown to increase glucose-stimulated insulin secretion and weight gain in mice (279). Administration of low concentrations of propionate reduced insulin sensitivity in mice and humans by stimulating glucagon and FABP4 production (280). Instead, butyrate promotes gut epithelial integrity (281), an anti-inflammatory milieu, resistance to enteropathogens and regulatory T cell generation. Further, butyrate can inhibit the epigenetic modulator histone deacetylases (HDACs) (282) and thereby induce an anti-inflammatory response particularly in intestinal cells (283, 284). Importantly, butyrate-producing bacteria are less abundant in fecal microbiota of T2D individuals (6), which might have deleterious effects on the intestinal immune system. Fiber intake is associated with a decreased risk for metabolic diseases via increased SCFAs production, however the mechanism and extend is still elusive.

### Trimethylamine-N-Oxide

Trimethylamine-N-oxide (TMAO) has attracted a lot of attention in the context of cardiometabolic diseases. TMAO is produced by the liver from trimethylamine (TMA), which on its turn is produced by intestinal microbiota from choline and carnitine-containing nutrients. TMAO has been linked to vascular inflammation (285, 286), e.g., via activation of NLRP3, and atherosclerotic plaque development, which could explain the increased risk for atherosclerotic cardiovascular disease and heart failure in people with higher levels of TMAO (287). Inhibition of the gut microbial TMA formation reduced macrophage foam cell formation and atherosclerotic lesions development in mice (288).

Further, a recent human trial found that energy-reducing diets decreased choline and L-carnitine. Those changes were associated with an improvement in fasting insulin and insulin resistance in overweight and obese adults. Further, there was a link between dietary fat intake and TMAO: increase in TMAO was related to lower improvements concerning glucose tolerance. All three metabolites (TMAO, choline and L-carnitine) were associated with changes in amino acids, in particular branched-chain (BCAAs) and aromatic amino acids (289) that have been associated with diabetes (290, 291) and changes in the gut microbiota (292).

### Bile Acids

Bile acids have been proposed to be involved in glucose metabolism (293). They include different molecules that are essential for nutrient absorption in the intestine and are transformed by the microbiota (293). Bile acids are end products from cholesterol that are formed in the liver and excreted in the intestine via the gall bladder. In the intestine, the bacteria transform primary into secondary bile acids (293). Further, bile acids have an effect on the microbiota composition, for example, cholic acids changed the gut microbiota and reduced systemic adiponectin levels in rats (294). Bile acids are efficiently absorbed in the enterohepatic circulation. Small parts leave the circulation and are either excreted in feces or end in the systemic circulation (293).

In the blood circulation, bile acids can bind to the farnesoid X receptor (FXR) and G protein coupled bile acid receptor 1 (TGR5), which are involved in glucose metabolism (295) and liver regeneration (296). Using an intestinal agonist for FXR induced amongst others GLP-1 secretion, change in the gut microbiota and improved glucose tolerance in mice (295). Antibiotic treatment reversed this metabolic phenotype (295), suggesting the gut microbiota to be involved. However, effects of FXR activation are rather contradictory. It seems to improve insulin sensitivity (297, 298), but also promotes obesity (299, 300). Knocking out the FXR receptor prevented obesity and metabolic changes in mice (301). Interestingly, FXR is expressed by various immune cells and is a modulator of the intestinal innate immunity and intestinal integrity (302). More studies are needed to disentangle the role of FXR in metainflammation.

Although these metabolites have given novel insight into the interaction between nutrient intake-gut microbiota composition—host metabolism and immunity, many data are derived from rodent models and require confirmation in humans. In addition, it remains to be proven that modulation of these metabolites in fact can alter metabolic disease and inflammation in humans.

## Interventions Aimed at Restoring Gut Microbiota Balance and Inflammation

Several interventions were partially able to restore gut microbiota symbiosis and associated metabolic function. In particular, dietary interventions have shown effects on microbiota function and meta-inflammatory effects. However, results from dietary interventions are complex and difficult to interpret since subjects show a low adherence and have high inter-individual variations. Therefore, other more specific approaches have been pursued to alter gut microbiota composition. Most commonly, supplementation of specific selective fermented ingredients that may alter microbiota composition (prebiotics) or supplementation of beneficial bacterial strains (probiotics) has been carried out, while in two studies, the metabolic effects of replacement of host microbiota by fecal microbiota transplantation (FMT) was investigated.

### Diet

Modulation of gut microbiota has been speculated to be a novel tool to improve the metabolic abnormalities associated with obesity and T2D. As was shown in a recent study, the success of a dietary intervention could be predicted based on baseline microbiota composition (151, 303). A higher microbial gene richness was associated with a lower inflammation (151). As diet is an important factor shaping the gut microbiota (304), dietary interventions (such as increasing plant-based fibers intake and reducing food additives such as artificial sweeteners) have been put forward as an attractive target to improve functional and compositional aspects of gut microbiota (146). A dietary intervention study which included whole grain products and prebiotics to particularly target the gut microbiota decreased typical pro-inflammatory bacteria belonging to *Enterobacteriaceae* taxa and lowered levels of LPS binding protein (LBP), several inflammatory markers and it increased systemic adiponectin concentrations (305) (Table 1). A meta-analysis shows that fiber intake leads to a higher abundance of *Bifidobacterium* spp. and *Lactobacillus* spp., which might contribute to an increase in fecal butyrate concentration (306). Although only associative in nature, these results suggest an effect of dietary intake on the gut microbiota and inflammatory read outs (305).

**Table 1:** Intervention studies that include the three way interaction (gut microbiota, inflammation, and glucose metabolism).

**Type**	**Intervention**	**Species**	**Gut microbiota**	**Inflammation**	**Glucose metabolism**	**Refernces**
Diet	Diet-induced weight-loss and weight-stabilization intervention	38 obese and 11 overweight humans (♂♀)	Gene richness ↑	hsCRP ↓	Weight ↓ HOMA-IR ↓ Fasting insulin ↓ Fasting glucose ↓	(151)
	Dietary scheme based on whole grains, traditional Chinese medicinal foods and prebiotics	93 obese humans (♂♀)	Enterobacteriaceae ↓ Desulfovibrionaceae ↓ Bifidobacteriaceae ↑ Gut permeability ↓	LBP ↓ TNF ↓ IL-6 ↓ Adiponectin ↑ CRP ↓	Weight ↓ Fasting glucose ↓ HOMA-IR ↓ HbA1c ↑	(305)
	Isocaloric low-carbohydrate diet with increased protein content	10 obese humans with NAFLD (♂♀)	Fecal SCFAs ↓ Folate-producing Streptococcus ↑	TNF ↓ IL-6 ↓	Weight ↓ Liver fat ↓ Fasting insulin ↓ HOMA-IR ↓	(307)
Prebiotics	Oligofructose enriched diet	Ob/ob and DIO mice	Firmicutes ↓ Bacteroidetes ↑ *A. muciniphila* ↑ Intestinal L-cells ↑	Plasma LPS ↓ Colonic IL-1 expression ↓	Weight ↓ Food intake ↓ Glucose tolerance ↑ GLP-1 ↑	(308)
	Oligofructose-enriched inulin or maltodextrin placebo	42 healthy children with overweight or obesity (♂♀)	Bifidobacterium spp. ↑ *Bacteroides vulgatus* ↓ Alpha diversity ↓	IL-6 ↓	Weight ↓ No differences in fasting glucose, insulin, or insulin resistance	(309)
	Daily oral Berberine	DIO rats (♂)	Diversity ↓ Fecal SCFA ↑ *Blautia* ↑ *Allobaculum* ↑	LBP ↓ MCP-1 ↓ Leptin ↓ Adiponectin ↑	Weight ↓ Food intake ↓ Fasting glucose ↓ Fasting insulin ↓ HOMA-IR ↓	(310)
	Oligofructose or placebo enriched diet	Ob/ob mice	Total bacteria ↑ *Bifidobacterium* spp ↑ *Lactobacillus* spp ↑ *Clostridium coccoides–Eubacterium rectale* cluster ↑ Gut permeability ↓ Intestinal tight junctions ↑	Plasma LPS ↓ Plasma Il-1α ↓ Plasma Il-1β ↓ Plasma MCP-1 ↓ Plasma TNF ↓ Plasma IFN-γ ↓ Plasma IL-6 ↓ Plasma IL-10 ↓	Portal GLP-1 ↑ Portal GLP-2 ↑	(311)
Probiotics	*Lactobacillus reuteri* or placebo	44 T2D humans (♂♀)	*L. reuteri* ↑ Serum deoxycholic acid↑ Serum unconjugated bile acids ↑	No changes	Insulin sensitivity ↑	(312)
	*Lactobacillus reuteri* or placebo	21 lean and obese glucose tolerant humans (♂♀)	*L. reuteri* ↑ No changes	No changes	Glucose stimulated GLP-1 ↑, GLP-2 ↑, insulin ↑, and C-peptide ↑ No changes in insulin sensitivity	(313)
	Single strain administration of *Lactobacillus paracasei* (LC), *Lactobacillus rhamnosus* (LR) or *Bifidobacterium animalis* (BA)	DIO mice (♂)	Shift toward lean microbiota type Cecal acetate (LC, LR) ↑	CLS ↓ Liver and adipose tissue TNF expression (BA) ↓ Serum LBP (BA) ↓	Weight ↓ HOMA-IR (LR) ↓ Glucose tolerance ↑	(314)
	*Lactobacillus rhamnosus* or placebo during weight loss and maintenance diet intervention	125 obese humans (♂♀)	Lachnospiraceae (♀) ↓	Leptin ↓	Weight (♀) ↓ No changes	(315)
	*Akkermansia muciniphila* or placebo	Ob/ob and DIO mice	Mucus thickness ↑ No compositional changes	Serum LPS ↓	Fasting glucose ↓ Glucose tolerance ↑	(316)
	*Akkermansia muciniphila* (pasteurized or live) or placebo	32 overweight and obese insulin-resistant humans (♂♀)	No changes	White blood cells (past.) ↓Serum LPS (past.) ↓	Fasting insulin (past.) ↓ Insulin resistance ↓	(317)
FMT	Metabolic syndrome (METS) or gastric bypass (RYGB) donor	22 metabolic syndrome humans (♂)	Fecal and serum bile acids (METS) ↑	Adipose expression of MCP-1 (RYGB) ↓	Insulin sensitivity (METS) ↓	(318)

*Summary of relevant findings that are discussed in this review*.

*hsCRP, highly sensitive C-reactive peptide; HOMA-IR, homeostatic model assessment for insulin resistance; LBP, lipopolysaccharide binding protein; TNF, tumor necrosis factor; IL, interleukin; NAFLD, Non-alcoholic fatty liver disease; DIO, diet induced obesity; MCP-1, Monocyte chemoattractant protein-1; SCFAs, short fain fatty acids; LPS, lipopolysaccharide; CLS, crown-like structure; FMT, fecal microbiota transplantation. ♂, male participants; ♀, female participants; Arrows, treatment leads to up or down regulation*.

Changes in diet, especially the amount of indigestible food components such as fibers change the gut microbiota composition (319). A low-fiber westernized diet was associated with decrease in beneficial Firmicutes bacteria and an increase in mucosa penetrating Proteobacteria (320). Further, several different types of fibers improved gut permeability and colitis in mice (304). The diversity is only partially restorable after reintroducing fibers into the diet, which leads to increasing permanent loss of bacterial strains in mice over multiple generations (152, 319). As a result of selection in favor of particular bacteria during western diet, this loss in diversity is mostly a loss of *Bacteroides*, known for their capacity to break down fibers (152, 320). This permanent loss of specialized bacteria may harbor vast consequences. Indeed, a rapid reduction in SCFAs was observed after low carbohydrate intake (307). Supplementation of butyrate directly, improved insulin sensitivity in mice (277), but failed in several human studies. Therefore, fibers are positively associated with the glucose tolerance, potentially via SCFAs production; however, the mechanism is still elusive.

### Prebiotics

Prebiotics have been described as selectively fermented ingredients that allow specific changes in the composition and/or activity of the gastrointestinal microflora that confers benefits upon host wellbeing and health (321). Current prebiotics are mainly complex carbohydrates, but also other compounds such as polyphenols and polyunsaturated fatty acids exert prebiotic effects (322). Next to improving stool consistency, prebiotics can be fermented to SCFAs that have various beneficial function on the host health (322).

Several prebiotics have been studied already in metabolic diseases. For example, oligofructose treatment in genetically obese mice, improved glucose homeostasis, inflammation and leptin sensitivity (308). Further, it increased L-cell content in rats with higher production of GLP-1 (323) and improved gut integrity, reduced endotoxemia as well as lowered inflammation in genetically obese mice (155). In humans, oligofructose-enriched inulin changed the gut microbiota in obese children, reduced body fat and decreased systemic IL-6 (309). Similarly, Berberine, a component of a Chinese herb, have shown beneficial effects on gut microbiota and glucose tolerance by increasing intestinal SCFA production and reducing inflammation in obese rats (310). A vast amount of animal studies suggests beneficial effect of prebiotics on the host glucose tolerance, however human studies fail to provide enough evidence (324). Further, only a few studies focused on inflammatory markers. More research is needed to assess the potential of prebiotic treatment to reduce metainflammation in humans.

### Probiotics

Probiotics are defined as live microorganisms which, when administered in adequate amounts, confer a health benefit on the host. The probiotic candidate must be a taxonomically defined. Further, safety and health benefits must be supported by reproducible human studies (325). When combined with prebiotics, they may be referred to as synbiotics. Given the associations between altered gut microbiota and T2D, and more specifically, a reduction in beneficial, butyrate producing bacteria, several trials have been performed in humans to improve metabolic health supplementing these bacteria, most commonly *Lactobacillus* or *Bifidobacterium* species (326).

In mice, *Lactobacillus reuteri* improved insulin sensitivity, gut permeability and aryl hydrocarbon receptor ligand production (327). Administration of a mixture of different *Lactobacillus* and *Bifidobacterium* strains improved glucose homeostasis, macrophage infiltration and changed the gut microbiota (314). Similarly, a multi-strain treatment (VSL#3) improved liver function, inflammation and insulin sensitivity in genetically obese mice (328). These rodent studies point out several beneficial roles of probiotics in metabolism and inflammation; however, probiotics are only moderately effective in humans.

The randomized clinical trials that were performed up-to-date however, were mostly of short duration and included a relatively small number of participants, and as such, generated contrasting effects. In an attempt to provide more clarity, several meta-analyses have been performed focusing on different outcomes. Two meta-analyses observed small improvements in fasting glucose levels (0.3–0.5 mmol/l) and reported conflicting results on fasting insulin levels or insulin sensitivity indices, dependent on the studies included in the meta-analysis given the significant heterogeneity (329, 330). In general, effects were more pronounced on glucose metabolism when interventions were performed in T2D patients (331, 332) as compared to normoglycemic obese participants and when a probiotic cocktail using several strains was administered instead of one. Other meta analyses reported no effect of probiotics on weight (333) and small improvements in total and LDL-cholesterol (329) with most benefits again in studies using multiple bacterial strains. The effects on systemic inflammation were only investigated in a handful of studies, where reductions in C-reactive protein (CRP), but not TNF were observed (329, 334).

*Akkermansia muciniphila* has been extensively studied in metabolic diseases (316) and has recently been tested in humans as a probiotic. Importantly, this strain inversely correlates with body weight in humans and rodents (316). First, administration of *A. muciniphila* in obese mice improved insulin sensitivity, metabolic endotoxemia and adipose tissue inflammation (316). Further, a sole purified membrane protein of that particular strain was able to improve metabolism and gut barrier function in obese as well as diabetic mice (335). Supplementation in humans improved insulin sensitivity and inflammation, however, the effects were rather small, but the authors concluded from this proof of principle study that the treatment is safe and has therapeutic potential (317).

A rather new strategy is the modification of exiting bacteria to produce biological active compounds. For example, in mice *L. reuteri* genetically modified to produce IL-22 improved liver function, inflammation and bacterial translocation (336). These findings give completely new treatment options that might be the future for the use of probiotic.

Thus, several studies point toward a beneficial effect of pre- and probiotics in people with metabolic diseases. However, the absolute improvements in clinically relevant outcomes such as fasting glucose levels or HbA1c have been very modest, especially when compared to the efficacy of commonly used glucose lowering agents. On the other hand, definitive conclusions cannot be drawn yet due to the small studies with short duration and heterogenic treatment groups. Further, it is still not clear whether probiotics colonize the gut during consumption (334). A recent study demonstrates that the colonization of probiotic depends on the host; thus, questioning the universal usage of probiotics and highlighting the importance of personalized medicine (337).

### Fecal Microbiota Transplantation

A drastic way to alter gut microbiota composition is by fecal microbiota transplantation (FMT). In this procedure, donor feces (obtained from a healthy donor following an extensive screening process) is transplanted into the recipient by upper GI (duodenal) infusion or lower GI (colonic) infusion. While anecdotally used in the past (338, 339), in more recent decades, the FMT procedure has become more common. More recently, two studies have been conducted to investigate whether transplantation of lean donor feces could improve glucose metabolism in obese metabolic syndrome patients. In a pilot study, lean donor FMT into nine obese males with metabolic syndrome induced a small but significant improvement in peripheral insulin sensitivity and a trend toward improved hepatic insulin sensitivity when compared to participants that underwent autologous gut microbiota infusion. Lean donor FMT resulted concomitantly in increased bacterial diversity and increased presence of butyrate producing bacteria (340).

The results from this pilot study were confirmed in a trial in 38 participants. Again, allogenic FMT improved insulin-stimulated glucose disposal in skeletal muscle after 6 weeks of treatment; this effect was most striking in patients with lower fecal microbiota diversity at baseline (341, 342). However, after 18 weeks, no effects could be observed on insulin sensitivity. Also, the microbiota composition had returned to the composition prior to the allogenic FMT (342). The mechanisms that underlie this beneficial effect remain to be investigated, as the FMT studies did not point to solid mechanisms responsible for these improvements. Thus, whether a reduction of bacterial translocation and/or low-grade inflammation contributes are involved remains hitherto unclear. The plasma levels of short-chain fatty acids such as butyrate were not altered.

However, a recent study compared FMTs from patients after bariatric surgery and patients with metabolic syndrome. Both donor feces were infused in people with metabolic syndrome. The latter decreased insulin sensitivity, along with an increase of several secondary bile acids (318). In contrast, the former led to a decrease in the macrophage attracting factor (MCP-1) in adipose tissue and in plasma. These results highlight the role of the gut microbiota in insulin tolerance and metabolic inflammation (318).

Future research in this area should focus on further standardization of the FMT technique (development of standard operating procedures) as well as validation in larger populations, including T2D patients. In addition, various refinements should be explored, including the transfer of specific strains (preferably in pill form) rather than feces, combination with prebiotics or antibiotics, and a better match between donors and receivers, thereby tailoring microbiota-based precision treatment. It is very likely that FMT into the upper GI tract might induce an inflammatory response since this part usually does not see fecal microbiota, however this has never been tested. Further, other microorganisms (e.g., viruses and fungi) than bacteria have been in the focus of gut microbiota related effects on host metabolism. Therefore, more studies are necessary to deceiver their role. Finally, the mechanisms that may drive the metabolic should be explored in depth.

## Conclusions and Future Perspectives

It is accepted already for several decades that an increased inflammatory tone has major influences on the glucose metabolism (60, 65). For example, expansion and infiltration of pro-inflammatory immune cells is present in several metabolic active tissues during the development of T2D (21, 92). This pro-inflammatory milieu has vast consequences on the organ function as seen in the development of insulin resistance (73), beta cell dysfunction (343), and fatty liver disease (344). The trigger or origin of this inflammatory response is still elusive. Only recently, we started to understand the role of the gut microbiota in those processes.

There is a great afford to identify and describe the underlying metabolic and inflammatory pathways; however, studies are often contradictory. Particularly, murine knock out models frequently give inconclusive results (216, 237, 298) and numerous inflammatory mediators have dual roles (93, 103, 228). Further, players of the immune system serve important physiological functions (other than purely inflammation) and have tissue specific responses (9, 345). These findings highlight that the immune system is a complex organization, which is often neither pro- nor anti-inflammatory *per se*. Additional, a controlled (acute) inflammatory response is important for the host to fight invading pathogens and remove damaged tissue. However, this resolution is disturbed in metabolic diseases leading to metainflammation. Environmental factors such as the diet (346) and genetics (347) appear to be the main drivers for those deviations. Lastly, research in metainflammation focuses on typical pro-inflammatory cytokines such as IL-6 and TNF, with less work done on anti-inflammatory mediators. Future treatment strategies could aim to increase anti-inflammatory cytokines in obese and diabetic humans.

To sustain a symbiotic relationship with the gut microbiota, a controlled and appropriate immune response is essential to benefit from the gut microbiota's numerous functions (348). Recent studies suggest a disturbed immune intestinal immune response in obesity (188). Further, the host influences the microbiota via the diet. By ingesting a diet rich in fiber, we promote the production of SCFAs that can improve the hosts energy homeostasis (275), glucose tolerance (277) and particularly the regulation of an adequate inflammatory response (349). Not only a reduced fiber intake (271), but also a genetically dependent lower butyrate update is present in T2D (164). Both functions, the gut microbiota (6) and the intestinal immune system (188), are disturbed in metabolic diseases. However, it is not clear which disturbance comes first.

Several potential mechanisms on how the microbiota influence glucose metabolism and inflammation have been described (114, 225, 247). Though, numerous contradictory reports make it difficult to make a clear conclusion (237, 280). Understanding housing techniques and the vivarium of animals are important for the research field (350). Further, lack of technical understanding might explain several contradictory findings in microbiota research. Omics and (bio) informatical strategies are getting more complex and difficult to grasp for the majority of scientists. Standardized and transparent protocols are important to move the field forward (351).

The concept of bacterial translocation is a good example for the lack of technical understanding (178, 179, 352). Although it is a reasonable concept, low amounts of bacteria in systemic tissue sides make it difficult to trust histology or sequencing techniques due to background noise. The identification of small bacterial metabolites is more plausible (114, 270, 353). We are only at the beginning to understand the effects of microbial metabolites on the host health.

Techniques to identify and describe microorganism are getting more affordable and available for the majority of research groups (354). Thereby, several bacterial species have been discovered and were associated with metabolic abnormalities (6, 143). Identification of single relevant strains is important to decipher mechanistic interactions, however the gut microbiota is a complex ecosystem with thousands of different groups of microorganisms. For example, the role of the virome (355) or mycobiome (356) is less explored. In that respect, a recent study highlight the influence of the fecal virome on glucose tolerance during FMT (355). More effort is needed to understand the whole microbial ecosystem.

Lastly, treatments targeting the gut microbiota to expand beneficial bacteria or exchange a “dysbiotic” microbiota with a symbiotic one show promising result. FMTs display moderate effects in metabolic diseases, however with a limited duration (342). Similarly, probiotic treatment only show moderate to no success (284). Individual microbiomes and responses to bacterial treatment are major challenges that have to be further evaluated (337, 342). Some of these findings might be explained by regional and ethnical differences (357, 358). Modifying existing bacteria might be a novel way to improve the response and help to regulate inflammation (336).

The field of microbiota research is very young with several challenges, but enormous potential. The complex relationship between millions of different microorganism, thousands host cell types and molecular mediators make it difficult to grasp the mechanism, but technical advantages are moving the field forward.

## Author Contributions

TS wrote the manuscript and prepared the figures. DR initiated the review. DR and HH supervised and revised the document. All authors discussed and contributed to the final manuscript.

## Conflict of Interest

MN is in the Scientific Advisory Board of Caelus Pharmaceuticals, the Netherlands. DR has acted as a consultant and received honoraria from Boehringer Ingelheim, Eli Lilly, Merck, Novo Nordisk and Sanofi and has received research operating funds from the Boehringer Ingelheim–Eli Lilly Diabetes Alliance, MSD, AstraZeneca and Novo Nordisk. BV was funded by the Canadian Institutes of Health Research, Crohn's and Colitis Canada, and the National Institutes of Health (R01AI134766). CV is supported by grants from the Canadian Institutes of Health Research (PJT - 153156) and JDRF (3-SRA-2014-39-Q-R), a grant from the National Institutes of Health. HH is supported by a senior fellowship (2019.82.004) of the Dutch Diabetes Research Foundation. The remaining authors declare that the research was conducted in the absence of any commercial or financial relationships that could be construed as a potential conflict of interest.
